# Editorial: Metabolism Linking Immunity and Inflammatory Phenotypes in Cardiovascular Disease

**DOI:** 10.3389/fcvm.2022.907530

**Published:** 2022-05-26

**Authors:** Murali Vijayan, Tharmarajan Ramprasath, Subbiah Ramasamy, Rajaraman D. Eri

**Affiliations:** ^1^Department of Internal Medicine, Texas Tech University Health Sciences Center, Lubbock, TX, United States; ^2^Center for Molecular and Translational Medicine, Georgia State University, Atlanta, GA, United States; ^3^Cardiac Metabolic Disease Laboratory, Department of Biochemistry, School of Biological Sciences, Madurai Kamaraj University, Madurai, India; ^4^College of Health and Medicine, School of Health Sciences, University of Tasmania, Launceston, TAS, Australia

**Keywords:** metabolism, inflammation, immunity, cardiovascular diseases, obesity

Cardiovascular diseases (CVD) are the primary public health concern. Inflammation is one of the major factors that involves in the development of many cardiovascular diseases through various mechanisms. These mechanisms are including endothelial dysfunction, inflammatory cell phenotype and their infiltration, proliferation of smooth muscle cells, etc. (1). CVD, is the most prevalent cause of mortality and morbidity in diabetic populations. Diabetes a metabolic disorder and the pathophysiology of both type 1 and type 2 diabetes (T1D and T1D) also linked with increased inflammation. As a result, diabetes has attracted growing interest in targeting inflammation to improve disease prevention and control (2). The accumulation of knowledge also indicates that metabolic disturbance contributes to the inflammatory process during the development of diabetes and heart diseases (3–6). Thus, linking inflammation to immune signaling pathways will establish a strong relationship of molecular metabolism and cardiovascular disease development. In recognition of the clinical value of this study, this subject featured six original research and six review papers in the field of metabolism-mediated inflammation and immune alterations with a larger perspective on cardiovascular and diabetic pathology.

In this special series of papers, Fang et al. described the mechanism of Slc39a2-mediated zinc homeostasis as well as the dynamic regulation of Slc39a2 during cardiac hypertrophy. The study also revealed a modulated innate immune signaling event during the phenylephrine-induced cardiomyocyte hypertrophy. The study concludes that targeting Slc39a2-intracellular zinc metabolism could be a novel strategy for treating heart diseases.

As apolipoproteins involve in binding and transporting lipid molecules to form lipoproteins, they have significant importance in many diseases, particularly cardiovascular disease events. To understand the polymorphic effect of these proteins, from the available data, Basavaraju et al. carried out gene cluster-based polymorphic studies on different APOE genes. The aim of this review was to discuss in details of the effects of various genetic polymorphisms of APOE genes and their impact in various health conditions, such as obesity, cardiovascular, stroke, Alzheimer's disease, diabetes, vascular complications, and other associated risks. This population genetic data has great insights into understanding the role of genetic polymorphism, correlated to several APOE protein-based medical risks.

Recent advances in gut microbiota research indicate that it is a new target for preventing and treating cardiovascular disease and diabetes. Using whole-genome shotgun sequencing technology, Chen et al. conducted a genome-wide association study on the gut microbiome of patients with Carotid atherosclerosis (CAS) and controls. Study found that the CAS group had significantly higher levels of several risk factors and inflammatory markers. This study also discovered that the majority of gut microbes and their pathways that were enriched in the CAS group had significant positive correlations with clinical characteristics. In addition, patients with CAS had higher levels of trimethylamine-N-oxide, which was mediated by a cyclic pathway, according to the study.

The role of heavy metal exposure, particularly cadmium (Cd), and its link to health risks has received increased attention in recent decades (7). The research of Zhang et al. identified chronic cadmium (Cd) mediated atherosclerosis (AS) *via* macrophage polarization. Their findings also revealed that receptor-interacting serine/threonine kinase 3 (RIPK3), *via* the Drp1 protein, regulated the Cd-induced mitochondrial homeostasis imbalance and increased the expressions of NF-κB-Nlrp3 inflammasome proteins. These molecular events eventually resulted in M1-type polarization of macrophages in the cardiovascular system, which aided in the development of AS. These findings revealed a novel mechanism for Cd-induced inflammation and provided new insights into the pathophysiology of heavy metal pollution-induced AS.

Velmurugan et al. aimed to investigate the prevalence of diabetes, cardiovascular risk factors, and its complications in rural farming and non-farming villages in Tamil Nadu, South India. The study, which was conducted among 0.1 million rural people in Tamil Nadu, South India, demonstrates the massive burden of diabetes in the rural world. The strong link between occupational agrochemical exposure and diabetes in rural communities highlights the need for change in diabetes clinical practice by focusing on occupational safety measures in agricultural policies.

Maternal obesity is undoubtedly a cause for concern that warrants further investigation, as interventions during this critical period may promote better health outcomes in the offspring. With rising obesity rates in the United States, it is critical to consider the long-term consequences for future generations. With the increasing concern of obesity rates witnessed in the United States, it is imperative to acknowledge the long-term effects on future generations. Shrestha et al. sought to review the risks of maternal obesity and to propose viable intervention strategies. Their data from human and animal studies revealed that nutritional interventions and physical activity could mitigate many of the negative effects of obesity on offspring metabolic health.

Coronavirus disease 2019 (COVID-19), caused by severe acute respiratory syndrome coronavirus 2 (SARS-CoV-2), is a global health emergency. Patients, who had COVID-19, displayed a variety of cardiovascular symptoms, during their post-recovery period. Hence to test if these vascular symptoms were caused by compromised endothelial barrier integrity, Raghavan et al. studied the relation between SARS-CoV-2 spike protein and host junctional proteins that maintain endothelial barrier integrity. According to the findings of the study, spike-induced degradation of endothelial junctional proteins affects endothelial barrier function, which further led to the vascular damage that is observed in COVID-19 affected people. He also reviewed the cardiovascular effects of COVID-19 infection in patients. This review displayed the most recent clinical reports of COVID-19-related cardiovascular complications on a large scale.

Wu et al. did a systematic review that offered information on the structure and physiological activities of Chemokine C-X-C motif ligand-1 (CXCL1). This review focuses on recent advances in research on the molecular mechanisms of CXCL1 in cardiac fibrosis. They also looked at the involvement of CXCL1 in the fibrosis of other organs. Furthermore, the research suggests that CXCL1 could be a key target to treat the cardiac fibrosis disease conditions.

Oh et al. conducted a meta-analysis of current clinical study data that assessed the relationship between the distribution of monocyte subsets in people with cardiometabolic disorders and cardiovascular disease (CVD) compared to healthy controls. They determined in this comprehensive study that persons with cardiometabolic diseases and CVD may have a larger percentage of intermediate (IM) and non-classical monocytes than healthy controls.

A meta-analysis done by Oh et al. evaluated the recent findings from clinical studies that examined the association between the distribution of monocyte subsets in subjects with cardiometabolic disorders and cardiovascular disease (CVD) compared to healthy controls. In this systematic review, they concluded that individuals with cardiometabolic disorders and CVD might have a higher percentage of intermediate (IM) and non-classical monocytes than healthy controls.

The goal of Song et al. was to find potential metabolic indicators in patients with acute coronary syndrome (ACS). They used UPLC-Orbitrap/MS system to conduct a metabonomics investigation on ACS serum samples. In serum samples from ACS patients, this metabolomics investigation discovered significantly altered metabolites and their pathways. Some of the candidates were found to have a strong diagnostic ability for ACS. They also discovered signaling pathways linked to ACS pathogenesis, providing a foundation for further research into the interaction between metabolites and ACS pathogenesis.

On the clinical translational front, Ma and Chen Summarized the current anti-inflammatory treatments' successes and unanswered questions for coronary atherosclerotic heart disease.

## Conclusion and Future Perspective

The collection of both research and systematic reviews presented in this special issue exhibits importance of metabolic immunity and inflammatory phenotypes in cardiovascular disease as well as and expands our understanding in this field. Through this knowledge, we hope that scientific community will be encouraged to address the remaining outstanding issues to advance scientific research on the interactions between immunity and cardiovascular disease. Furthermore, the identification of comparable and consistent local and systemic interactions would pave the road for improved understanding of the disease pathology and medical management.

## Author Contributions

All of the contributors contributed equally to the editing and writing. Furthermore, all authors gave their approval to the final edition.

## Conflict of Interest

The authors declare that the research was conducted in the absence of any commercial or financial relationships that could be construed as a potential conflict of interest.

## Publisher's Note

All claims expressed in this article are solely those of the authors and do not necessarily represent those of their affiliated organizations, or those of the publisher, the editors and the reviewers. Any product that may be evaluated in this article, or claim that may be made by its manufacturer, is not guaranteed or endorsed by the publisher.

## References

[1] Ramprasath T Han YM Zhang D Yu CJ Zou MH. Tryptophan catabolism and inflammation: a novel therapeutic target for aortic diseases. Front Immunol. (2021) 12:731701. 10.3389/fimmu.2021.731701 34630411 PMC8496902

[2] Tsalamandris S Antonopoulos AS Oikonomou E Papamikroulis GA Vogiatzi G Papaioannou S. The role of inflammation in diabetes: current concepts and future perspectives. Eur Cardiol. (2019) 14:50–9. 10.15420/ecr.2018.33.1 31131037 PMC6523054

[3] Arnold N Lechner K Waldeyer C Shapiro MD Koenig W. Inflammation and cardiovascular disease: the future. Eur Cardiol. (2021) 16:e20. 10.15420/ecr.2020.50 34093741 PMC8157394

[4] Chait A Den Hartigh LJ. Adipose tissue distribution, inflammation and its metabolic consequences, including diabetes and cardiovascular disease. Front Cardiovasc Med. (2020) 7:22. 10.3389/fcvm.2020.00022 32158768 PMC7052117

[5] Wu H Ballantyne CM. Metabolic inflammation and insulin resistance in obesity. Circ Res. (2020) 126:1549–64. 10.1161/CIRCRESAHA.119.315896 32437299 PMC7250139

[6] Battineni G Sagaro GG Chintalapudi N Amenta F Tomassoni D Tayebati SK. Impact of obesity-induced inflammation on cardiovascular diseases (CVD). Int J Mol Sci. (2021) 22. 10.3390/ijms22094798 33946540 PMC8125716

[7] Xu P Liu A Li F Tinkov AA Liu L Zhou JC. Associations between metabolic syndrome and four heavy metals: a systematic review and meta-analysis. Environ Pollut. (2021) 273:116480. 10.1016/j.envpol.2021.116480 33486246

